# Supplementary material for: HSPA5 Promotes the Proliferation, Metastasis and Regulates Ferroptosis of Bladder Cancer
Source: Int J Mol Sci. 2023 Mar 7;24(6):5144. doi: 10.3390/ijms24065144 (PMC10048805; doi:10.3390/ijms24065144)
Supplement: Supplementary file 1 [file ijms-24-05144-s001.zip › ijms-2195736-supplementary/ijms-2195736-SI/Figure S1-2.pdf]

supplementary data：

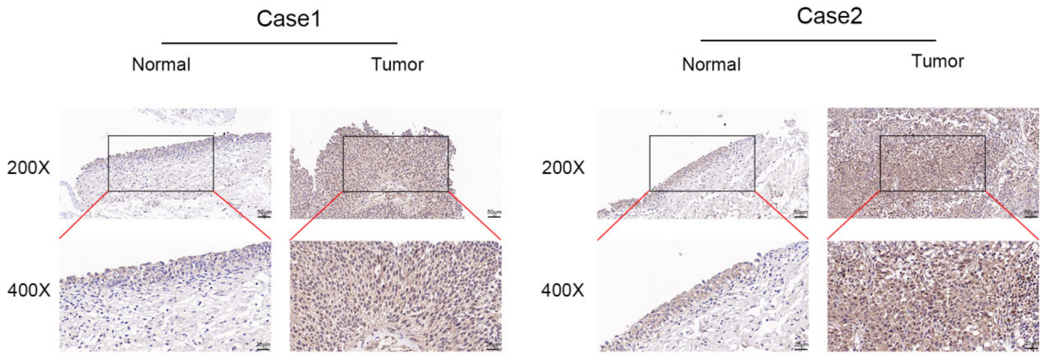

Figure S1. VEGFA expression in clinical samples.

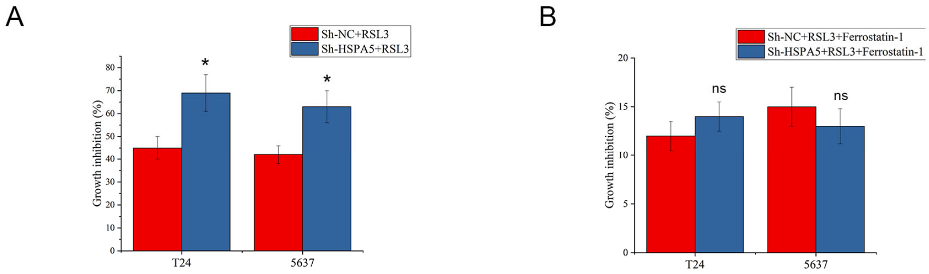

Figure S2. HSPA5 inhibition increased BCa cell sensitivity to ferroptosis. (A) HSPA5 downregulation increased BCa cell sensitivity to RSL3. (B) The growth inhibition after treatment with RSL3 and Ferrostatin-1 in Sh-NC and Sh-HSPA5 groups. .
