# Supplementary material for: HSPA5 Promotes the Proliferation, Metastasis and Regulates Ferroptosis of Bladder Cancer
Source: Int J Mol Sci. 2023 Mar 7;24(6):5144. doi: 10.3390/ijms24065144 (PMC10048805; doi:10.3390/ijms24065144)
Supplement: Supplementary file 1 [file ijms-24-05144-s001.zip › ijms-2195736-supplementary/ijms-2195736-SI/Table S1.pdf]

name	gender	age	hospitalization number	diagnosis	date	surgical modalities	pathological diagnosis	pathology number	first confirmed diagnosis
Qiusheng,Tong	male	57	10068014	Bladder mass	2020/2/21	TURBT	Low-grade noninvasive papillary urothelial carcinoma	202006659	Yes
Hanqiao,Tan	male	56	10075666	bladder cancer	2020/10/21	TURBT	Low-grade noninvasive papillary urothelial carcinoma	202030646	Yes
Wancehng,chen	male	67	10075641	Multiple bladder masses	2020/10/19	Radical Cystectomy	Low-grade noninvasive papillary urothelial carcinoma	2020030647	Yes
jiawen,chen	male	57	10080400	Bladder foreign body	2020/11/3	Radical Cystectomy	High-grade invasive papillary urothelial carcinoma	202034180	Yes
chuanyan,li	male	76	10083866	haematuria	2020/11/10	TURBT	Low-grade noninvasive papillary urothelial carcinoma	202034691	Yes
guangsheng,chen	male	66	10085458	bladder cancer	2020/11/22	TURBT	Low-grade invasive papillary urothelial carcinoma	202036269	Yes
liansu,yang	male	32	10089696	Bladder mass lesions	2020/11/24	TURBT	Low-grade noninvasive papillary urothelial carcinoma	202037132	Yes
pansheng,yu	male	67	10088194	nephrolithiasis	2020/11/30	Radical Cystectomy	High-grade invasive papillary urothelial carcinoma	202037615	Yes
wenjun,gong	male	76	10106851	bladder cancer	2021/1/22	TURBT	High-grade invasive papillary urothelial carcinoma	202102701	Yes
yang,pang	female	81	10107553	bladder cancer	2021/1/22	TURBT	High-grade invasive papillary urothelial carcinoma	202102702	Yes
fuchu,xia	male	59	10107824	Hydronephrosis	2021/1/26	Radical Cystectomy	Low-grade noninvasive papillary urothelial carcinoma	202102949	Yes
shuo,liu	male	77	10122518	New organisms of the bladder neck	2021/3/6	Radical Cystectomy	High-grade invasive papillary urothelial carcinoma	202108754	Yes
yuanxiang,zhou	male	80	10127000	bladder cancer	2021/3/22	Radical Cystectomy	High-grade invasive papillary urothelial carcinoma	202111371	Yes
qigu,zhou	male	70	10161301	bladder cancer	2021/6/11	Radical Cystectomy	High-grade invasive papillary urothelial carcinoma	202128531	Yes
minglan,xia	female	76	10109352	haematuria	2021/9/6	Radical Cystectomy	High-grade invasive papillary urothelial carcinoma	202146645	Yes
yongchu,deng	male	73	10197119	Foreign bodies in the bladder	2021/9/11	Radical Cystectomy	Low-grade invasive papillary urothelial carcinoma	202147446	Yes
wanshou,li	female	63	10248978	bladder cancer	2021/12/27	Radical Cystectomy	High-grade invasive papillary urothelial carcinoma	202169851	Yes
